# Supplementary material for: Trash to Treasure: How Insect Protein and Waste Containers Can Improve the Environmental Footprint of Mosquito Egg Releases
Source: Pathogens. 2022 Mar 18;11(3):373. doi: 10.3390/pathogens11030373 (PMC8950251; doi:10.3390/pathogens11030373)
Supplement: Supplementary file 1 [file pathogens-11-00373-s001.zip › pathogens-1637245-SM.pdf]

Table S1. Mosquito strains and corresponding experiments.

Experiment	Mosquito Line
Diet and waste container lab experiments	Aus *w*Mel
Kiribati field test	Kiribati wild-type

Table S2. Liquid diet feeding regime followed per 150 larvae.

Days Post-hatch	Number of Tetramin Tablets	Amount of Liquid Food (beef liver powder-, cricket- or black soldier fly-based)
0	0.5 tablets	4.13 μL
1	0	413 μL in morning and 413 μL in evening
2	0	413 μL
3	0	1.65 mL
4	0.5 tablets	1.65 mL
5	0	1.23 mL
6	0	413 μL
7	0.25 tablets	413 μL

Table S3. qPCR primer and probe sequences. Reference gene *Ribosomal Protein S17 (RpS17)* and target gene *Wolbachia surface protein (wsp)* [44].

Primer Name	Sequence
Rps17_TaqM_Probe	CAGGAGGAGGAACGTGAGCGCAG (FAM-BHQ1)
wspTM-LC640	LC640-TCCTTTGGAACCCGCTGTGAATGA-iowaBlack
wspTM-2F	CATTGGTGTTGGTGTTGGTG
wspTM-2R	ACACCAGCTTTTACTTGACCAG
Rps17_TaqM_FW	TCCGTGGTATCTCCATCAAGCT
Rps17_TaqM_RV	CACTTCCGGCACGTAGTTGTC

Table S4. Statistical indicators for all statistical tests.

Figure	Measurement	Statistical test	Degrees of Freedom	F-statistic	Chi-squared	P-value
1.a	Emergence rate	ANOVA	3	4.0625	na	0.0253
1.b	Wing Length	ANOVA	3	6.985	na	0.0253
1.c	Wolbachia density	Kruskal-Wallis	na	na	3.131	0.3719
2.a	Hatch rate - Fly test	ANOVA	1	3.3569	na	0.0794
2.a	Hatch rate - Cricket test	ANOVA	1	1.3843	na	0.2509
2.b	Emergence rate - Fly test	ANOVA	1	6.0118	na	0.0219
2.b	Emergence rate - Cricket test	ANOVA	1	0.581	na	0.4634
2.c	Wing length - Fly test	Mann-Whitney	na	na	na	0.2268
2.c	Wing length - Cricket test	Mann-Whitney	na	na	na	0.7719
2.d	Wolbachia density - Fly test	Mann-Whitney	na	na	na	0.0151
2.d	Wolbachia density - Cricket test	Mann-Whitney	na	na	na	5.65×10^{-14}
3.a	Emergence rate	ANOVA	3	4.0625	na	0.0253
3.b	Wing Length	ANOVA	3	6.985	na	0.0003
3.c	Wolbachia density	Kruskal-Wallis	4	na	8.3471	0.0797
4.a	Emergence rate	ANOVA	4	0.8384	na	0.5249
4.b	Mosquito death	ANOVA	4	0.542	na	0.708
S2.a	Emergence rate	Kruskal-Wallis	4	na	1.4943	0.8276
S2.b	Wing Length	ANOVA	4	1.475	na	0.218
S2.c	Wolbachia density	Kruskal-Wallis	4	na	8.3356	0.08

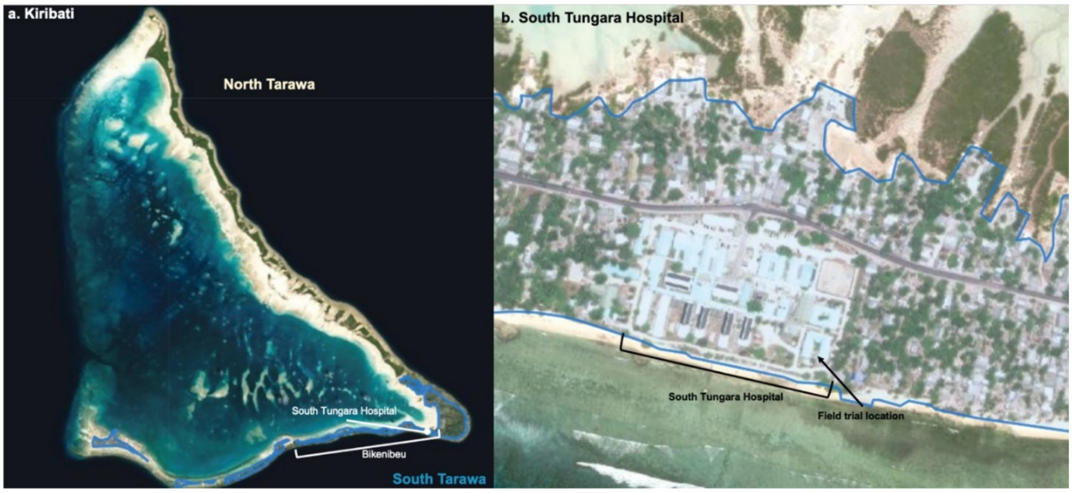

Figure S1. Map of field trial location **a.** Kiribati, North and South Tarawa, and **b.** South Tungara hospital.

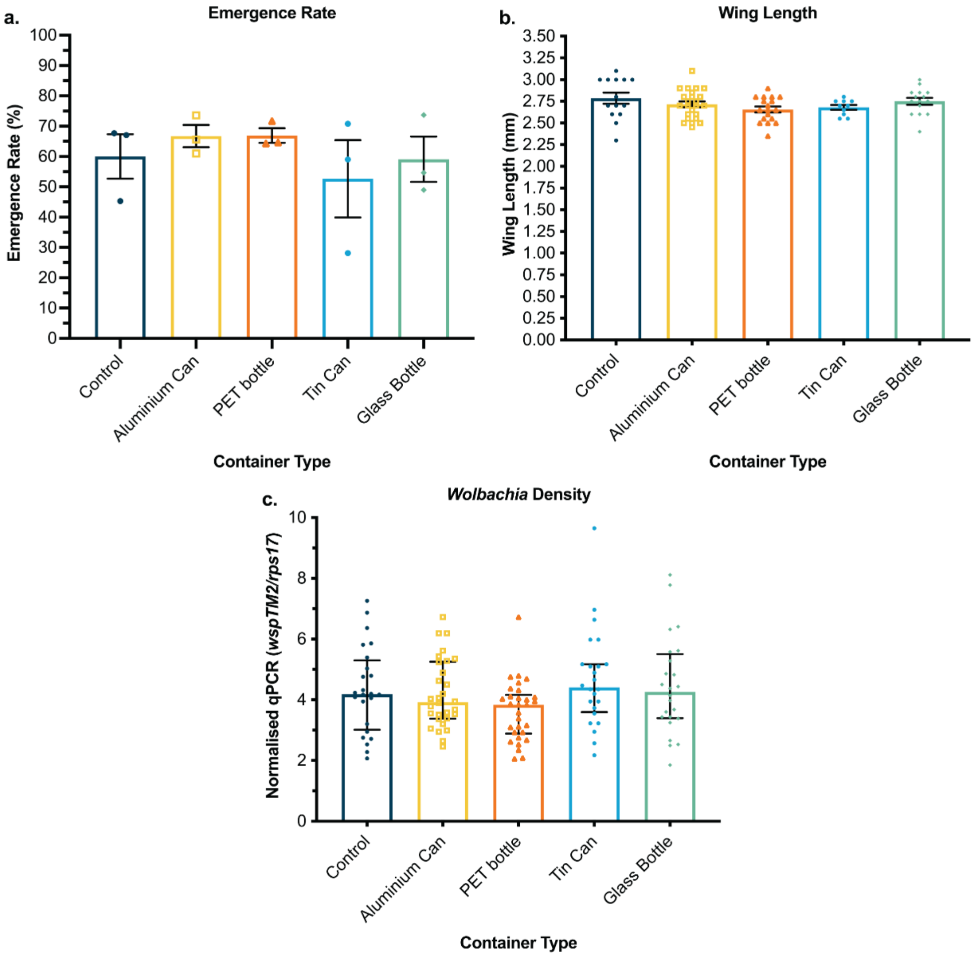

Figure S2. Development and adult fitness comparison between *w*Mel-infected *Ae. aegypti* mosquitoes reared in five different containers. Aus *w*Mel *Ae. aegypti* eggs were fed cricket-based diet inside of capsules. **(a)** Emergence rate **(b)** Wing length and **(c)** *Wolbachia* densities are shown. Each data point represents one cup (emergence), one wing from an individual mosquito (wing length) or one mosquito (*Wolbachia* density). Ordinary one-way analysis of variance was performed on the dataset, followed by Tukey's pairwise comparison test, or Kruskal-Wallis test for Wolbachia density. No significant differences were detected. Error bars represent standard error of the mean.

Strategy 1: Aluminium cans, distributed through churches (community-led)

	Steps	Staff	Vehicles
1	Purchase containers from Kiribati recycling company	0	0
2	Transport containers from recycling facility to onsite office location (Tungara Central Hospital)	4	2
3	Purchase labels and place on containers	2	0
4	Distribute cans and capsules to churches for distribution amongst the community	6	3
5	Conduct community engagement activities to reinforce messaging and check correct implementation	2	1
6	Transport capsule fortnightly to churches for distribution to the community	4	2
7	After completion, inform participants, via the church, to recycle cans	1	0

Strategy 2: Cardboard containers, distributed through churches (community-led)

	Steps	Staff	Vehicles
1	Purchase and import cardboard containers into Kiribati	0	0
2	Distribute containers and capsules to churches for distribution amongst the community	6	3
3	Conduct community engagement activities to reinforce messaging and check correct implementation	2	1
4	Transport capsule fortnightly to churches for distribution to the community	4	2
5	After completion, inform participants, via the church, to dispose of containers in landfill waste	1	0

Strategy 3: Aluminium cans, distributed directly to the community (staff-led)

	Steps	Staff	Vehicles
1	Purchase containers from Kiribati recycling facility	0	0
2	Transport containers from recycling facility to onsite office location (Tungara Central Hospital)	4	2
3	Purchase labels and place on containers	2	1
4	Distribute cans and capsules directly to houses, completing 5 rounds of releases over 10 weeks	18	9
5	During final releases, inform participants that they can recycle their cans	1	0

Strategy 4: Cardboard containers, distributed directly to the community (staff-led)

	Steps	Staff	Vehicles
1	Purchase and import cardboard containers into Kiribati	0	0
4	Distribute containers and capsules directly to houses, completing 5 rounds of releases over 10 weeks	18	9
5	During final releases, inform participants that they can dispose of containers in landfill waste	1	0

Figure S3. End-to-end process for each release strategy, including staff and vehicle requirements.
